# The relationship between cardiorespiratory fitness and indices of fat mass and fat-free mass in adults

**DOI:** 10.3389/fspor.2025.1583432

**Published:** 2025-07-24

**Authors:** Collin J. Popp, Elliot D. Jesch

**Affiliations:** ^1^ Department of Population Health, Institute for Excellence in Health Equity, NYU Langone Health, New York, NY, United States; ^2^ Department of Food, Nutrition and Packaging Sciences, Clemson University, Clemson, SC, United States

**Keywords:** aerobic fitness, body composition, body fat, lean mass, physical activity

## Abstract

**Purpose:**

Cardiorespiratory fitness (CRF) independently predicts cardiovascular disease risk and mortality. In addition, body composition levels characterized by excess adiposity (fat mass) and low levels of fat-free mass (FFM) are strongly associated with poor health status. The relationship between CRF and body composition, especially indices adjusted for height [fat mass index (FMI), fat-free mass index (FFMI)] has not been well established in otherwise healthy adults.

**Methods:**

A sample (*n* = 82) of adults completed measures for body composition using dual-energy x-ray absorptiometry and estimated V˙O_2max_ by way of an Åstrand-Rhyming submaximal cycle ergometer test. Total daily energy expenditure (TDEE) and moderate-to-vigorous physical activity (MVPA) were measured using a SenseWear Armband activity monitor. The associations between body composition (FMI, FFMI), and CRF were examined using multiple linear regression models adjusting for sex (model 1) and TDEE (model 2).

**Results:**

Participants were young (age: 24 ± 9 year), 64% female, with a BMI of 25.4 ± 4.9 kg·m^−2^. The mean absolute and relative estimated V̇O_2max_ were 3.02 ± 1.0 L·min^−1^ and 42.1 ± 12.2 ml·kg^−1^·min^−1^, respectively. FMI was negatively associated CRF (L·min^−1^) in model 2 (*B* = −0.106, 95%CI −0.16, −0.05, *p* < 0.001) but not model 1 (*B* = −0.011, 95%CI −0.03, 0.01, *p* = 0.271). FFMI was positively associated with CRF (L·min^−1^) (model 1: *B* = 0.087, 95%CI 0.03, 0.14), *p* = 0.004) but not after adjusting for TDEE (model 2: *B* = 0.026, 95%CI −0.07, 0.12, *p* = 0.585).

**Conclusion:**

FM adjusted for height (FMI), but not fat-free mass (FFMI), is negatively associated with cardiorespiratory fitness in adults.

## Introduction

A mounting body of evidence has shown that excess levels of adiposity are associated with insufficient levels of physical activity (PA) and increased sedentary behavior (1, 2). The coupling of insufficient PA levels and evaluated obesity rates resulted in the reduction of cardiorespiratory fitness (CRF), the ability to perform large muscle, dynamic, moderate-to-vigorous intensity exercise for prolonged periods. CRF is a strong predictor of all-cause mortality in healthy adults (3, 4), and older adults, independent of total adipose tissue levels (5). A meta-analysis found a dose-response relationship by which for every 1-metabolic equivalent of task (MET) higher level of CRF was associated with an 11%–17% reduction in all-cause mortality (6). The relationship between CRF and obesity is often neglected in routine health assessment, despite the strong relationship between the two (7, 8). Furthermore, low CRF may be an early indicator of insulin resistance in individuals at risk for type 2 diabetes (9).

Studies indicate there is a negative relationship between body mass index (BMI) and CRF, whereby excess body weight is associated with lower CRF (10–12). However, the utility of BMI as an accurate measure of adiposity is constant under scrutiny. The use of gold-standard methods for body composition analysis, such as dual-energy x-ray absorptiometry (DEXA), provides more accurate and precise methods of body composition, especially fat-free mass (FFM). However, prior studies often only focus on total FFM and fat mass (FM) and exclude indices of body composition and height, called FFM index (FFMI) and FM index (FMI). Adjusting FM and FFM for height is important as height takes into account body size as prior evidence suggests taller individuals have greater lung volume (13, 14); therefore, height may contribute to an individual's fitness levels (15). Additionally, prior studies examining the relationship between body composition and CRF often neglect to adjust for physical activity levels using wearable devices (16). Therefore, this study aims to determine the relationship between submaximal CRF and body composition indices (FFMI and FMI) in adults. Previously, Goran et al. reported that FFM was the strongest determinant of aerobic capacity (V̇O_2max_). Additionally, after adjusting for FFM, and FM does not impair maximal aerobic capacity (17). Therefore, based on these findings by Goran et al., we hypothesizes that FFMI, will be positively associated with CRF but not FMI.

## Materials and methods

### Overall study design

The participants were healthy males and females recruited via flyers, e-mail, and word-of-mouth as volunteers. Participants were included if they were adults between the ages of 18 and 69, not pregnant, lactating or attempting to become pregnant or have any condition that precludes them from wearing a band on their left arm (e.g., left breast mastectomy). All participants completed and signed both a physical activity readiness questionnaire (PAR-Q) and written informed consent before partaking in research activities. Those who answered “yes” to any line on the PAR-Q were required to obtain written consent from their physician to participate. Anyone unwilling to sign the informed consent was excused from the study. The Institutional Review Board (IRB) approved all study procedures for human participants. Informed consent was obtained from all participants included in the study.

### Anthropometrics

Participants had their height and body weight (BW) were measured using a SECA 763 digital scale and stadiometer (SECA North America, Chino, CA, USA) in minimal athletic attire. Prior to the height and weight measurements, participants removed their shoes and any excess clothing or jewelry. Height was measured to the nearest 0.1 cm and body weight was measured to the nearest 0.1 kg. We calculated body surface area (BSA) using the DuBois equation (18). Whole-body dual x-ray absorptiometry (DEXA) scans were performed using a Hologic Discovery QDR Series (Hologic, Inc., Bedford, MA, USA) densitometer to measure body composition. The densitometer was calibrated daily using a whole-body phantom. The Hologic software provides measures of whole-body and regional fat mass (FM), lean mass, percent body fat (BF), and bone mass. We used the two-compartment model of body composition to include FM and FFM. FFM was calculated by subtracting the total BW from FM. FMI and FFMI are defined as the mass of fat and lean tissue relative to height in meters squared, respectively. Resting heart rate (RHR), systolic (SBP), and diastolic blood pressure (DBP) were measured after a 5-minute resting period using an automated blood pressure machine (WelchAllyn, Skaneateles Falls, NY, USA).

### Cardiorespiratory fitness and physical activity

After completing measures for body composition, participants completed a submaximal cycle ergometer test to estimate aerobic capacity (V̇O_2max_). Participants were fitted with a Polar heart rate monitor (Polar Electro Inc., Lake Success, NY, USA) around the thoracic region just below the sternum. A Monark 828 cycle ergometer (Monark Exercise AB, Vansbro, Sweden) was used for the Astrand-Rhyming cycle ergometer test to measure predicted V̇O_2max_ (19). Participants were instructed to pedal at 50 revolutions per minute (RPM). During the 6-minute protocol, bike resistance increased every 3 min so the participants' heart rate was between 125 and 170 beats per minute (BPM). If the participant's heart rate was not within that range after 6 min, bike resistance increased again, and the test was extended by 3 min. The test continued, with resistance increasing every 3 min, until the participant's heart rate was within range for two consecutive minutes. Once the participant reached the target heart rate range, the resistance declined, and the test ended. The participant then pedaled for another 1–3 min to cool down. Results from the test were collected via the MicroFit HealthWizard 5 software (MicroFit Inc., Fresno, CA, USA).

Physical activity and total daily energy expenditure (TDEE) were assessed with the SenseWear Armband (SWA, BodyMedia Inc., Pittsburgh, PA). The armband uses minute-by-minute measurements of tri-axial accelerometry, galvanic skin response, skin temperature, heat flux, and near-body temperature to estimate energy expenditure using proprietary regression models (Version 7.0 professional) (16). The SWA provides accurate estimates of TDEE and physical activity of free-living adults (20, 21). All participants were instructed to wear the SWA continuously for 7 days, only removing the SWA when bathing/showering or swimming. Adherence to wearing the SWA was defined as duration on the body of ≥20 h/day (85% daily adherence). Less than 10% (*n* = 8) of participants fell below the 20-hour cutoff for SWA data; their SWA data was not included in the final analysis. The average wear time for the participants who were adherent to the SWA wear time (*n* = 74) was 1,383.4 ± 54.5 min·d^−1^ (∼23 h·d^−1^).

### Statistical analysis

Data are reported as mean ± standard deviation (SD) unless otherwise stated. An independent samples *t*-test was run to compare demographics between sexes. Pearson correlation coefficients were calculated to examine the relationships between anthropometrics (e.g., FMI) and CRF separated by sex. A linear regression analysis was conducted using aerobic capacity, reported in absolute (L·min^−1^) and relative (ml·kg^−1^·min^−1^) terms, as the dependent variable, and body composition (BW, BSA, BF, FM, FFM, FMI, FFMI) as the independent variables (22). We recognize that the traditional expression of CRF is in relative terms (ml·kg^−1^·min^−1^), which adjusts for body weight; however, analyzing relative CRF and body composition (kg) may overinflate the explained variance between body composition and CRF (16). Therefore, we will focus our analysis on comparing body composition variables to absolute CRF (L·min^−1^) but report the comparisons with relative CRF (ml·kg^−1^·min^−1^) in the supplemental material. Two models were developed. Model 1 included sex and anthropometric measures (i.e., BMI, FFMI), and model 2 included the variables in model 1 with the addition of total daily energy expenditure (TDEE, kcal·d^−1^), as TDEE correlates with CRF (*r* = 0.733, *p* < 0.001).

We did not perform an *a priori* power calculation as part of the study. However, a *post-hoc* power analysis was conducted using G*Power 3.1 to determine the observed power with an effect size of 0.15 (medium), an alpha level of 0.05, and either two predictors (*n* = 80; Model 1: sex and anthropometrics) or three predictors (*n* = 74; Model 2: Model 1 + TDEE). There was an 86.9% power in Model 1 and 78.3% power in Model 2 to detect a true effect, respectively. Collinearity tolerance and variance inflation factor (VIF) statistics were calculated for each model. Multicollinearity exists when the variance inflation factor (VIF) is greater than 5–10 and the tolerance is lower than 0.2, respectively (23). All statistical analyses were completed in SPSS (Version 23, IBM SPSS), and all figures were generated using GraphPad Prism (Version 10.3.1, GraphPad Software, LLC, Boston, MA, USA).

## Results

The final sample included eighty adults, and all the participants were non-smoking. The CRF data for two participants were missing; therefore, they were excluded from the final analysis. Additionally, less than 10% (*n* = 8) of participants fell below the 20-hour cutoff for SWA data, and their SWA data were excluded from the final analysis (Model 2). The average wear time for the participants who adhered to the SWA wear time (*n* = 74) was 1,383.4 ± 54.5 min·d^−1^ (∼23 h·d^−1^). Table 1 describes the demographics of all participants, separated by sex. On average, the sample was young, mostly female, and considered overweight (BMI > 25.0 kg/m^2^). Comparing males and females, there was no difference for age (*p* = 0.28), DBP (*p* = 0.43), BSA (*p* = 0.55), ST (*p* = 0.18), and TST (*p* = 0.23). BMI was not significantly different (*p* = 0.20) between males and females; however, as expected, males had lower BF (*p* < 0.001), FM (*p* = 0.02), and FMI (*p* < 0.001), and higher FFM (*p* < 0.001) and FFMI (*p* < 0.001) compared to females. Males were more active, with a significantly greater TDEE (*p* < 0.001), AEE (*p* < 0.001), and MVPA (*p* = 0.01) compared to females. These findings are corroborated by higher CRF [both absolute (*p* < 0.001) and relative (*p* = 0.01)] and lower RHR (*p* = 0.002) in males compared to females.

**Table 1:** Demographics and anthropometrics in the full sample and by sex.

Variable	All (*n* = 82)		Males (*n* = 29)		Females (*n* = 53)		*P* value males vs. females
	Mean ± SD	Range	Mean ± SD	Range	Mean ± SD	Range	
Age, years	24 ± 9	17.0–60.0	26 ± 10	18–59	23 ± 9	17–60	0.28
Height, cm	168.63 ± 11.5	114.6–199.6	178.6 ± 8.7	166.1–199.6	163.3 ± 8.9	114.6–178.3	**<0.001**
Weight, kg	72.75 ± 16.8	46.6–135.5	83.1 ± 1.8	63.1–105.0	67.2 ± 16.5	46.6–135.5	**<0.001**
BMI, kg·m^−2^	25.3 ± 5.0	18.0–46.5	26.1 ± 3.5	21.4–35.9	24.8 ± 5.5	18.0–46.5	0.20
RHR, bpm	73.89 ± 13.0	47.0–112.0	67.6 ± 10.4	50.0–90.0	77.3 ± 3.2	47.0–112.0	**0.002**
SBP, mmHg	116.58 ± 12.5	92.0–156.0	122.9 ± 11.2	106.0–156.0	113.2 ± 11.8	92.0–151.0	**0.002**
DBP, mmHg	75.25 ± 8.5	60.0–98.0	74.2 ± 7.5	60.0–87.0	75.8 ± 8.9	60.0–98.0	0.43
Absolute CRF, L·min^−1^	3.02 ± 1.0	1.3–5.7	3.8 ± 0.93	1.97–5.73	2.6 ± 0.69	1.3–4.3	**<0.001**
Relative CRF, ml·kg^−1^·min^−1^	42.1 ± 12.2	19.1–83.20	46.5 ± 10.0	23.3–67.8	39.8 ± 12.7	19.1–83.2	**0.01**
BF, %	28.8 ± 8.6	9.8–49.6	21.5 ± 5.2	15.4–35.7	32.7 ± 7.4	9.8–49.6	**<0.001**
FM, kg	21.3 ± 9.2	10.8–63.3	18.1 ± 6.2	11.2–35.5	23.1 ± 10.2	10.8–63.3	**0.02**
FFM, kg	52.1 ± 12.9	32.2–82.5	65.4 ± 8.7	46.0–82.5	44.7 ± 8.0	32.2–74.2	**<0.001**
FMI, kg·m^−2^	7.6 ± 3.4	3.6–21.7	5.7 ± 2.0	3.6–10.8	8.5 ± 3.6	4.6–21.7	**<0.001**
FFMI, kg·m^−2^	17.1 ± 3.0	12.4–31.4	20.5 ± 2.3	16.7–25.5	16.8 ± 3.2	12.4–31.4	**<0.001**
FFM:FM	2.7 ± 1.1	1.0–5.5	3.8 ± 0.97	1.8–5.5	2.1 ± 0.61	1.0–3.8	**<0.001**
BSA (m^2^)	1.8 ± 0.23	1.2–2.4	2.0 ± 0.16	1.7–2.3	1.7 ± 0.20	1.2–2.4	0.55
TDEE, kcal·d^−1^a^^	2,698.6 ± 638.3	1,637.0–4,489.0	3,315.3 ± 489.3	2,519.0–4,499.0	2,328.6 ± 377.6	1,637.03,216.0	**<0.001**
AEE, kcal·d^−1^a^^	706.3 ± 413.4	38.0–2,499.0	989.4 ± 455.3	249.0–2,499.0	536.4 ± 273.0	38.0–1,386.0	**<0.001**
ST, min·d^−1^a^^	1,257.8 ± 74.2	716.0–1,406.0	1,245.6 ± 66.5	945.0–1,345.0	1,265.1 ± 78.3	716.0–406.0	0.18
MVPA, min·d^−1^a^^	125.8 ± 58.3	6.0–270.0	149.7 ± 55.9	41.0–255.0	111.5 ± 55.5	6.0–270.0	**0.01**
TST, min^a^	384.2 ± 45.3	180.0–522.0	392.3 ± 48.0	274.0–522.0	379.2 ± 43.4	180.0–459.0	0.23

Bold indicates significance values, *p* < 0.05. Mean ± SD, [Range]; BMI, body mass index; BSA, body surface area; RHR, resting heart rate; SBP, systolic blood pressure; diastolic blood pressure; CRF, cardiorespiratory fitness; BF, body fat; FFM:FM, fat-free mass-to-fat mass ratio; FFM, fat-free mass; FFMI, fat-free mass index; FM, fat mass; FMI, fat mass index; TDEE, total daily energy expenditure; AEE, active energy expenditure; ST, sedentary time; TST, total sleep time; MVPA, moderate-to-vigorous physical activity.

^a^ Sense wear Armband (SWA) includes those who were adherent (≥20 h/day, 85% daily adherence): *n* = 74.

Pearson correlations indicate significant associations between anthropometrics and aerobic fitness by sex. We found significant positive correlations between BW and BSA in males but not females (Table 2). Notably, we found a negative association between FMI and CRF in males (*r* = −0.411, *p* = 0.01) but not females (*r* = −0.014, *p* = 0.21). In contrast, there was a positive correlation between FFMI and CRF in females (*r* = 0.357, *p* = 0.005) but not males (*r* = 0.293, *p* = 0.06) despite a positive correlation between FFM and CRF for both sexes.

**Table 2:** Pearson correlations between body composition and absolute cardiorespiratory fitness by sex.

Absolute CRF (L·min^−1^)		
Variable	Pearson correlation coefficient	*P* value
BW (kg)		
Males	0.418	**0.01**
Females	0.162	0.12
BMI (kg/m^2^)		
Males	−0.040	0.42
Females	0.091	0.26
BSA (m^2^)		
Males	0.611	**<0.001**
Females	0.177	0.10
BF (%)		
Males	−0.557	**0.001**
Females	−0.415	**0.001**
FM (kg)		
Males	−0.256	0.09
Females	−0.080	0.29
FFM (kg)		
Males	0.733	**<0.001**
Females	0.431	**<0.001**
FMI (kg/m^2^)		
Males	−0.411	**0.01**
Females	−0.114	0.21
FFMI (kg/m^2^)		
Males	0.293	0.06
Females	0.357	**0.005**

Bold indicates significance values, *p* < 0.05. BW, body weight; BSA, body surface area; BF, body fat; BMI, body mass index; CRF, cardiorespiratory fitness; FFM, fat-free mass; FFMI, fat-free mass index; FM, fat mass; FMI, fat mass index; Males: *n* = 28; Females: *n* = 53.

**Figure 1:**
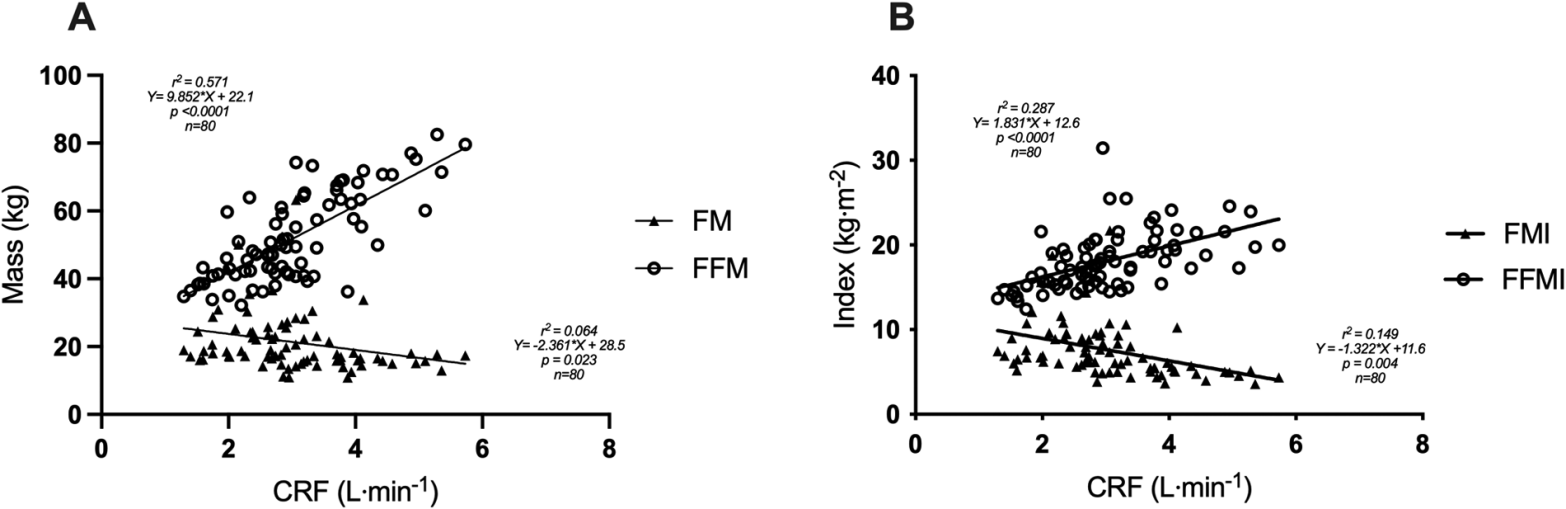
Associations between body composition and absolute cardiorespiratory fitness. Unadjusted models showed FFM was positively associated with absolute CRF, but not FM **(A)**. Neither FMI nor FFMI was associated with absolute CRF **(B)**. CRF, cardiorespiratory fitness; FFM, fat-free mass; FFMI, fat-free mass index; FM, fat mass; FMI, fat mass index. *Two participants did not have aerobic fitness levels or body composition measures. *n* = 80.

In the unadjusted models, absolute CRF was significantly associated with BW (*r*^2^ = 0.199, *p* < 0.001), BSA (*r*^2^ = 0.336, *p* < 0.001), and BF (*r*^2^ = 0.432, *p* < 0.001), but not BMI (*r*^2^ = 0.012, *p* = 0.329). The unadjusted linear regression models between FM, FFM, and absolute CRF are shown in Figure 1. Notably, absolute CRF was positively associated with FFM (*r*^2^ = 0.571, *p* < 0.001) and negatively associated with FM (*r*^2^ = 0.064, *p* = 0.023) (Figure 1A). In this unadjusted model, 57.1% of the variance in absolute CRF is explained by FFM, while FM accounts for only 6.4% of the variance in absolute CRF. Considering height as a contributing factor to CRF, in the unadjusted regression models, FMI continued to be negatively associated with absolute CRF, with FMI accounting for nearly double the variance in CRF (14.9%) compared to FM (Figure 1B). Additionally, FFMI was positively associated with absolute CRF, although accounting for height reduced the variance to 28.7%. Unadjusted models between anthropometrics and relative CRF (ml·kg^−1^·min^−1^) are shown in the supplemental material (Supplementary Figure S1).

No collinearity was indicated between the dependent and independent variables, except for FFM in model 2 (tolerance = 0.156, VIF = 6.4). Table 3 details adjusted linear models examining the associations between absolute CRF and body composition. For both BW and BSA, in model 1, there was a significant association with absolute CRF; however, neither BW nor BSA was associated with CRF after adjusting for TDEE (model 2). The opposite was true for the relationship between BMI and CRF when adjusting for TDEE, resulting in a significant negative correlation (*p* = 0.018). FM was not associated with absolute CRF (model 1), but there was a significant negative association between FM and absolute CRF in model 2. The relationship between FM and CRF was similar when taking into account height, as FMI was only negatively associated with CRF in model 2, but not model 1 (*p* = 0.101). In both models, FFM was positively associated with absolute CRF; however, multicollinearity exists with model 2, and the results should be interpreted with caution. Interestingly, FFMI was not significantly associated with absolute CRF when adjusting for TDEE, despite a positive association between FFMI and CRF in model 1.

**Table 3:** Association between body composition and cardiorespiratory fitness.

Absolute CRF (L·min^−1^)			
Variable	*B* (95%CI)	*β*	*P* value
BW (kg)			
Model 1	0.013 (0.001, 0.024)	0.210	**0.035**
Model 2	−0.008 (−0.025, 0.008)	−0.122	0.324
BMI (kg/m^2^)			
Model 1	0.008 (−0.029, 0.044)	0.038	0.679
Model 2	−0.050 (−0.090, −0.009)	−0.212	**0.018**
BSA (m^2^)			
Model 1	1.382 (0.487, 2.28)	0.327	**0.003**
Model 2	0.177 (−1.185, 1.539)	0.039	0.796
BF (%)			
Model 1	−0.052 (−0.076, −0.028)	−0.447	**<0.001**
Model 2	−0.059 (−0.083, −0.035)	−0.475	**<0.001**
FM (kg)			
Model 1	−0.011 (−0.031, 0.009)	−0.102	0.271
Model 2	−0.036 (−0.056, −0.016)	−0.290	**<0.001**
FFM (kg)			
Model 1	0.053 (0.035, 0.071)	0.690	**<0.001**
Model 2^a^	0.047 (0.016, 0.077)	0.592	**0.003**
FMI (kg/m^2^)			
Model 1	−0.047 (−0.103, 0.009)	−0.161	0.101
Model 2	−0.106 (−0.161, −0.052)	−0.324	**<0.001**
FFMI (kg/m^2^)			
Model 1	0.087 (0.029, 0.145)	0.296	**0.004**
Model 2	0.026 (−0.068, 0.120)	0.074	0.585

Bold indicates significance values, *p* < 0.05. BW, body weight; BSA, body surface area; BF, body fat; BMI, body mass index; CRF, cardiorespiratory fitness; FFM, fat-free mass; FFMI, fat-free mass index; FM, fat mass; FMI, fat mass index; Linear regression model 1 (*n* = 80): Sex; Linear regression model 2 (*n* = 74): Model 1 + TDEE; Sense wear Armband (SWA) includes those who were adherent (≥20 h/day, 85% daily adherence): *n* = 74.

^a^ Collinearity for FFM in model 2 (tolerance = 0.156, VIF = 6.4).

## Discussion

The evidence linking CRF and body composition, specifically height-adjusted indices of FM and FFM, is scarce despite the association between body fat and aerobic fitness. In this sample, we assessed body composition and submaximal aerobic fitness in otherwise healthy, non-smoking adults. Contrary to our hypothesis, FMI was negatively associated with absolute CRF when adjusting for TDEE. Our findings suggest that higher FM may have a negative influence on CRF, regardless of energy expenditure. Additionally, while FFM was associated with absolute CRF in both models, FFMI was not significantly associated with absolute CRF after adjusting for additional covariates (sex, TDEE). Considering FFMI is a size-adjusted measure of FFM, our findings suggest that height influences both FFM and CRF.

Considering that FFM is a key determinant of energy expenditure and an important predictor of physical activity level, it's no surprise that prior studies have shown FFM to be a determinant of aerobic fitness (13, 14, 16, 17, 19, 24). Goran et al. reported nearly a quarter century ago that FM does not affect CRF, specifically V̇O_2max_ (17). Specifically, they measured women before and after weight loss and found V̇O_2max_ relative to FFM (ml·kg FFM^−1^·min^−1^) was not different after losing weight; however, V̇O_2max_ relative to body weight (i.e., relative V̇O_2max_) was significantly lower after weight loss. V̇O_2max_ relative to FFM provides a specific “physiological status” of the cardiorespiratory system, to which CRF does not seem to be influenced by excess body fat. Additionally, Batterham et al. found a linear relationship between lean mass and V̇O_2peak,_ but not between total body mass and V̇O_2_peak, in 1,304 adult men (24). Finally, a study by Yanek et al. (*n* = 191) examined cross-sectionally CRF and body composition using DEXA in a sample of predominantly African American adults and found that both fat mass and lean mass were determinants of CRF (16). However, lean mass was the strongest positive predictor of absolute CRF, explaining 27% of the variance in men and 21% of the variance in women. The results from our study align with prior studies that demonstrate a linear relationship between FFM and aerobic fitness, independent of sex.

Despite FFM being a key determinant of CRF, prior studies also show FM is a predictor of V̇O_2max_ (16, 25–27). The mixed results of FFM and FM contributing to aerobic fitness are likely due to methodological issues. For example, prior studies show differences in the estimate of body composition by bioelectrical impedance compared to DEXA (28, 29). Additionally, assessment of CRF using either treadmill testing compared to cycle ergometer testing impacts the degree of V̇O_2max_. For example, using a cycle ergometer testing negates the impact of body weight on aerobic testing in adults with overweight and obesity, whereas in treadmill testing, adults with overweight or obesity may achieve greater V̇O_2max_ (30).

Our findings are insightful, given that prior studies exclude body size measures by incorporating body composition indices, such as FMI and FFMI. A rather interesting finding was the negative correlation between FMI and CRF. Similar findings have been reported elsewhere when examining FMI and CRF. A cross-sectional study of over 300 adults (>35 year) found a negative relationship between CRF and FMI; however, the relationship was only in men and not women (25). One might argue that losing excess body fat is more advantageous for metabolic health compared to increasing lean mass. A study of healthy adult monozygotic and dizygotic twins found stronger associations between metabolic health, such as insulin sensitivity and liver fat, and FMI rather than CRF or FFMI (31). This suggests excess adiposity may have a greater impact on metabolic health compared to non-fat tissue, irrespective of genetic or environmental determinants.

Our findings also point to the importance of scaling FM and FFM by height. It has been well-documented in primates and humans that bone and muscle mass vary across individuals of different body size (e.g., stature) (32, 33). For example, Heymsfield et al. assessed whole-body and regional body composition in the large NHANES cohort. The authors demonstrated that taller individuals tend to have higher proportions of bone and skeletal muscle within their total body mass, with the most significant contributions coming from the lower extremities (32). As a result, this may result in greater aerobic fitness. However, a higher proportion of bone and skeletal muscle mass in the lower extremities may not necessarily translate to greater CRF. Predictors of CRF beyond body composition include muscle capillary density, muscle fiber type, and oxidative enzymes (i.e., cytochrome c oxidase) (34).

Our study has several strengths. We measured body composition using DEXA, which is considered the gold standard measure of body composition. We used a wearable device to objectively determine TDEE and physical activity, rather than relying on self-report or questionnaires. Despite these findings, we recognize there are limitations. In model 2, our *post-hoc* power calculation was slightly underpowered (78%), which runs the risk of a Type II error. We detected multicollinearity in model 2, which includes FFM and CRF, due to the strong correlation between FFM and TDEE. Multicollinearity can inflate the standard errors of estimated coefficients, making it more challenging to accurately determine the individual effect of each predictor (23). Our sample was young, rather healthy, and mostly White, limiting the generalizability of our findings to other populations. There are limitations to the use of the two-compartment model of body composition (FM and FFM) as the two-compartment model assumes known proportions of FFM, as water, protein, and mineral are constant (35). However, total body water can vary within-person as a result of physical activity and daily fluid intake, which may result in a less accurate measure of FFM (36). We did perform a submaximal exercise test rather than a maximal treadmill test, which may have deviated from the true V̇O_2max_ (37). Due to the study design's cross-sectional nature, we cannot determine the cause-and-effect relationship between FMI, FFMI, and CRF in our sample.

In conclusion, our primary findings indicate that body adiposity adjusted for height (FMI), but not fat-free tissues (e.g., FFMI), is negatively associated with cardiorespiratory fitness in young, non-smoking adults. Moreover, the negative relationship between FMI and CRF after adjusting for energy expenditure suggests a potential independent influence of an individual's adipose tissue levels on CRF. In future trials, we encourage researchers to examine the relationship between body composition indices and CRF using more precision methods, such as with the four-compartment model of body composition (bone mineral content, body volume, total body water, body mass). Additionally, future trials should examine the functional characteristic of adipose tissue in relationship with CRF, and the potential negative feedback mechanisms that higher adiposity may have at a molecular and cellular level on CRF and physical activity. From a clinical standpoint, improvements in CRF should be a key focus on improving metabolic health as sustained increase in physical fitness reduces cardiometabolic risk, and should be included in lifestyle interventions, especially with individuals seeking weight loss (38). Clinicians, dietitians, and exercise physiologists should encourage individuals to maintain a healthy body weight and regularly perform moderate-to-vigorous physical activity to maintain an optimal CRF.

## Data Availability

The raw data supporting the conclusions of this article will be made available by the authors, without undue reservation.
