# Supplementary material for: The relationship between cardiorespiratory fitness and indices of fat mass and fat-free mass in adults
Source: Front Sports Act Living. 2025 Jul 24;7:1583432. doi: 10.3389/fspor.2025.1583432 (PMC12337484; doi:10.3389/fspor.2025.1583432)
Supplement: Supplementary file 1 [file Supplementaryfile1.docx]

Supplemental Material

Title: The relationship between cardiorespiratory fitness and indices of fat mass and fat-free mass in adults.

Author Names: Collin J. Popp, PhD, MS, RD and Elliot D. Jesch, PhD

Corresponding author:

Collin J Popp

180 Madison Ave.

New York, NY 10016; 7-12

New York University Langone Health;

Institute for Excellence in Health Equity, Center for Healthful Behavior Change,

Department of Population Health

Collin.popp@nyulangone.org

Office: (646) 501-3427

**Unadjusted models**

In the unadjusted models, relative CRF was significantly associated with BMI (r^2^=0.175, p<0.001) and %BF, (r^2^=0.480, p<0.001), but not BW (r^2^=0.041, p=0.07) and BSA (r^2^=0.005, p=0.534). Unadjusted models for FM, FMI, FFM and FFMI with relative CRF are shown in sFigure 1.

**sTable 1: Table 2: Association between CRF and body composition indices**

|  | Relative CRF (ml∙kg^-1^∙min^-1^) | | |
| --- | --- | --- | --- |
|  | B (95%CI) | β | P |
| BW (kg) |  |  |  |
| Model 1 | -0.297 (-0.463, -0.131) | -0.406 | **<0.001** |
| Model 2 | -0.584 (-0.828, -0.340) | -0.732 | **<0.001** |
| BMI (kg/m^2^) |  |  |  |
| Model 1 | -1.122 (-1.600, -0.644) | -0.456 | **<0.001** |
| Model 2 | -1.643 (-2.254, -1.033) | -0.589 | **<0.001** |
| BSA (m^2^) |  |  |  |
| Model 1 | -18.927 (-32.489, -5.366) | -0.366 | **0.007** |
| Model 2 | -37.635 (-59.058, -16.212) | -0.685 | **<0.001** |
| BF (%) |  |  |  |
| Model 1 | -1.231 (-1.516, -0.946) | -0.866 | **<0.001** |
| Model 2 | -1.326 (-1.676, -0.976) | -0.894 | **<0.001** |
| FM (kg) |  |  |  |
| Model 1 | -0.778 (-1.018, -0.538) | -0.591 | **<0.001** |
| Model 2 | -0.996 (-1.285, -0.707) | -0.665 | **<0.001** |
| FFM (kg) |  |  |  |
| Model 1 | -0.047 (-0.368, 0.274) | -0.050 | 0.771 |
| Model 2 | -0.094 (-0.645, 0.456) | -0.100 | 0.733 |
| FMI (kg/m^2^) |  |  |  |
| Model 1 | -2.251 (-2.941, -1.561) | -0.629 | **<0.001** |
| Model 2 | -2.706 (-3.502, -1.911) | -0.688 | **<0.001** |
| FFMI (kg/m^2^) |  |  |  |
| Model 1 | -0.069 (-0.986, 0.848) | -0.019 | 0.881 |
| Model 2 | -1.152 (-2.744, 0.441) | -0.276 | 0.154 |

BW, body weight; BSA, body surface area; BF, body fat; BMI, body mass index; CRF, cardiorespiratory fitness; FFM, fat-free mass; FFMI, fat-free mass index; FM, fat mass; FMI, fat mass index; Linear regression model 1 (n=80): Sex; Linear regression model 2 (n=74): Model 1 + TDEE; Sense wear Armband (SWA) includes those who were adherent (≥20 hr/d, 85% daily adherence): n=74; Significance is in bold; p<0.05

**sFigure 1: Association between body composition and cardiorespiratory fitness relative to body mass.**


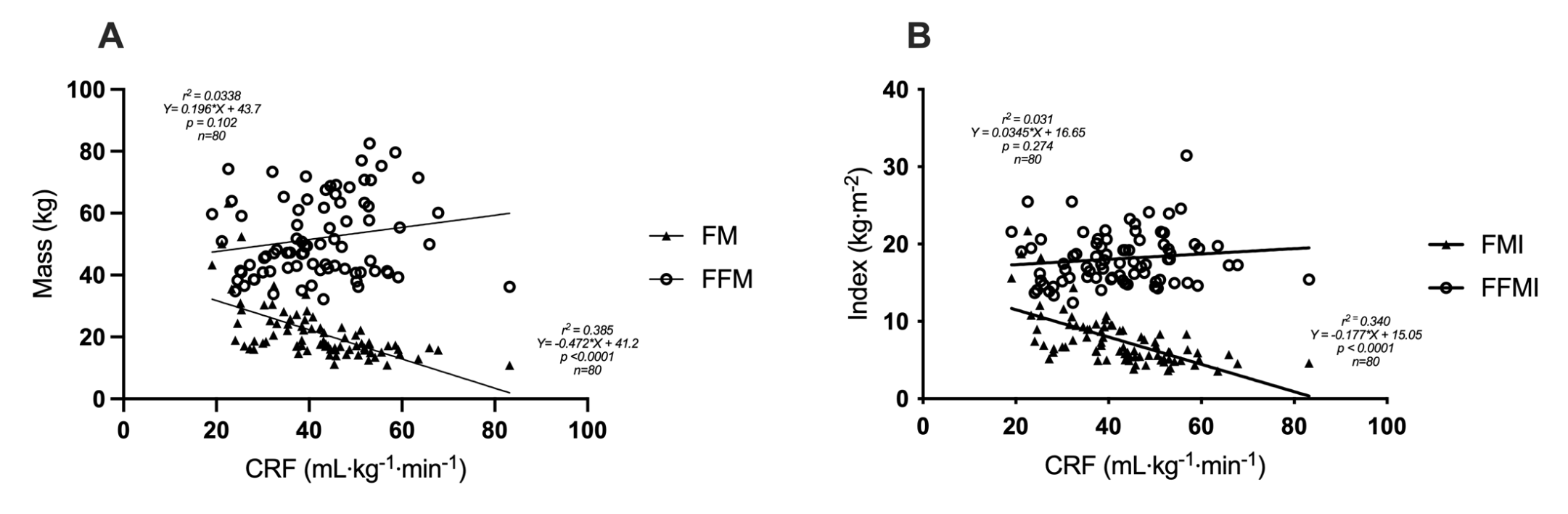


Unadjusted models showed FFM was positively associated with relative CRF and FM was negatively associated relative CRF (Panel A). Adjusting for height, FMI was negatively associated with releative CRF but not FFMI (Panel B). CRF, cardiorespiratory fitness; FFM, fat free mass; FFMI, fat free mass index; FM, fat mass; FMI, fat mass index

*Two participants did not have aerobic fitness levels or body composition measures. n=80.
